# Melatonin as an Anti-Inflammatory Agent Modulating Inflammasome Activation

**DOI:** 10.1155/2017/1835195

**Published:** 2017-10-01

**Authors:** Gaia Favero, Lorenzo Franceschetti, Francesca Bonomini, Luigi Fabrizio Rodella, Rita Rezzani

**Affiliations:** ^1^Anatomy and Physiopathology Division, Department of Clinical and Experimental Sciences, University of Brescia, Viale Europa 11, 25123 Brescia, Italy; ^2^Interdepartmental University Center of Research “Adaption and Regeneration of Tissues and Organs (ARTO)”, University of Brescia, Brescia, Italy

## Abstract

Inflammation may be defined as the innate response to harmful stimuli such as pathogens, injury, and metabolic stress; its ultimate function is to restore the physiological homeostatic state. The exact aetiology leading to the development of inflammation is not known, but a combination of genetic, epigenetic, and environmental factors seems to play an important role in the pathogenesis of many inflammation-related clinical conditions. Recent studies suggest that the pathogenesis of different inflammatory diseases also involves the inflammasomes, intracellular multiprotein complexes that mediate activation of inflammatory caspases thereby inducing the secretion of proinflammatory cytokines. Melatonin, an endogenous indoleamine, is considered an important multitasking molecule with fundamental clinical applications. It is involved in mood modulation, sexual behavior, vasomotor control, and immunomodulation and influences energy metabolism; moreover, it acts as an oncostatic and antiaging molecule. Melatonin is an important antioxidant and also a widespread anti-inflammatory molecule, modulating both pro- and anti-inflammatory cytokines in different pathophysiological conditions. This review, first, gives an overview concerning the growing importance of melatonin in the inflammatory-mediated pathological conditions and, then, focuses on its roles and its protective effects against the activation of the inflammasomes and, in particular, of the NLRP3 inflammasome.

## 1. Melatonin

Melatonin (N-acetyl-5-methoxytryptamine) is an endogenous indoleamine widely distributed in plants, unicellular organisms, algae, bacteria, invertebrates, and vertebrates [1–3]. In vertebrates, circulating melatonin is largely derived from the pineal gland [2, 4–6], although there are other organs such as gastrointestinal tract, epithelial hair follicles, skin, retina, salivary glands, platelets, and lymphocytes that produce melatonin [5, 7–11]. Specialized photoreceptive cells in the retina detect a restricted bandwidth of visible light; this information projects directly to the suprachiasmatic nucleus (SCN), the central circadian pacemaker, that triggers the pineal gland to produce this indoleamine during darkness [6, 12–14]. The maximal melatonin plasma concentration occurs usually 3–5 hours after darkness onset, and its level during the daily light period is low or even undetectable [15, 16].

The synthesis of melatonin is a multistep process, which starts from the essential aromatic amino tryptophan that is picked up from the blood circulation and hydroxylated, by tryptophan hydroxylase, in 5-hydroxytryptophan (5-HTP). 5-HTP is converted to serotonin by the aromatic amino acid decarboxylase, and serotonin is subsequently converted into N-acetylserotonin (NAS) by the enzyme arylalkylamine N-acetyltransferase. The final step of melatonin synthesis is the conversion of NAS to melatonin by hydroxyindole-O-methyl transferase [3, 17–19]. Pineal melatonin is immediately released into the blood stream in a circadian manner in response to the above reported photoperiodic information received via the retinopineal pathway [20–22]. Interestingly, most of the extrapineal organs, except for the retina, may not produce melatonin in a circadian manner and it is not normally released into the blood stream in any significant amount [7, 21, 23]. In these organs, melatonin presumably functions mainly as an antioxidant to protect cells from oxidative damage [7, 14, 24].

There are three major known pathways of melatonin degradation: (a) the classical hepatic catabolic pathway that generates 6-hydroxymelatonin that is then excreted via the kidney as a sulphate conjugate [3, 5, 25, 26]; (b) the alternative indolic pathway that produces 5-methoxyindole acetic acid or 5-methoxytryptophol [27, 28]; and (c) the kynuric pathway that produces N^1^-acetyl-N^2^-formyl-5-kynuramine (AFMK) [29–31]. In addition to the antioxidant properties of melatonin, AFMK and N^1^-acetyl-5-methoxykinuramine (AMK) are two important melatonin metabolites that have excellent radical scavenging activity [5, 32, 33].

The first identified function of melatonin was skin-lightening property observed in fish and amphibian [34, 35]. Subsequently, it has been shown that melatonin is a multitasking molecule with many functions, including the involvement in sleep initiation, mood modulation, sexual behavior, vasomotor control, and immunomodulation. Moreover, melatonin has anti-inflammatory and antioxidant actions influencing energy metabolism [36–38] and it acts as an oncostatic and antiaging molecule [3, 21, 30, 36, 39–42]. Thus, melatonin is considered an important multitasking molecule with fundamental clinical applications [14]. Melatonin's actions may be mediated by an interaction with specific membrane-bound receptors [3, 43, 44] or they may be receptor-independent; the latter mainly relates to its direct radical scavenging functions [9, 45]. Melatonin receptors include membrane and nuclear binding sites [7, 43]. As of 2011, two membrane-bound melatonin receptors were identified and characterized (MT1 and MT2), which are G protein-coupled seven transmembrane receptors [3, 46–48]. MT1 receptors are expressed in the retina, ovary, testis, mammary gland, gallbladder, liver, kidney, immune cells, exocrine pancreas, and cardiovascular system [5, 30, 49], whereas, MT2 receptors are expressed in the duodenal enterocytes, immune system, hypothalamus, SCN, retina, pituitary, blood vessels, testes, kidney, gastrointestinal tract, mammary glands, adipose tissue, and skin [3, 50–52]. Furthermore, MT1 melatonin receptors are related mainly to reproductive, metabolic, and vasoconstrictive functions, whereas MT2 receptors are involved in the control of circadian rhythms, dopamine release in the retina, and vasodilatation [7, 49]. Furthermore, melatonin may also bind to the MT3 receptor (quinone reductase II), which, however, does not fulfill the criteria of a classical melatonin membrane receptor; it is thought to be a molecular target for antimalarial drugs [53, 54] and seems involved in regulating intraocular pressure [43, 55]. Although the MT3 receptor has not yet been found in humans, it is highly expressed in the liver and kidneys and, in moderate amounts, in the heart, adipose tissue, and brain of hamster [3, 56, 57].

Nuclear binding sites have also been identified for melatonin, and in fact, melatonin might function also through orphan receptors from the retinoid orphan receptor (ROR) and retinoid Z receptor family [43, 58]. ROR*α* receptors are ubiquitously expressed in all mammalian tissues, and high levels were detected in lymphocytes, neutrophils, and monocytes, while ROR*β* subform is expressed in the brain, pineal gland, retina, and spleen [59–61]. Melatonin may also interact with cytosolic proteins including calmodulin and calreticulin, which are involved in the cytoskeleton regulation and control of nuclear receptors, respectively [7, 62, 63].

Alterations of the 24-hour melatonin profile may be associated with a large variety of pathologies [18]. In fact, endogenous melatonin was observed significantly lower in breast, lung, and prostate cancer patients [64–66]. This reduction in melatonin levels was observed also in some psychiatric disorders, like schizophrenia and depression, in which the nighttime melatonin level is lower with respect to healthy person [66–68]; also in patients suffering from metabolic syndrome, diabetes, or sleep disorders, the melatonin rhythm timing is modified [66, 69–72]. Furthermore, in recent decades, humans have altered the natural light-dark cycle by increasing light at night and spending most of their time indoors; these lifestyle factors induce the disorganization of the circadian system and cause chronodisruption [73], including perturbations of the melatonin rhythm. The reduction in melatonin is associated with gradual losses in antioxidant protection and immunological and anti-inflammatory effects. In fact, epidemiological studies showed that chronodisruption is associated with an increased incidence of cardiovascular diseases, diabetes, obesity, cognitive and affective impairments, premature aging, and some types of cancer [74]. Throughout life, melatonin levels are also significantly reduced; in young children, nocturnal melatonin levels are highest [66, 75]. During aging, the amplitude of the melatonin peak and total melatonin production is significantly reduced, although there is a great interindividual variability regarding these mechanisms [76, 77]. It is important to underline also that higher melatonin levels likely have important and positive roles in healthy aging and longevity [56, 66]. In fact, a reduction in endogenous melatonin levels throughout life seems to cause greater organism detriments [7, 78]. It is important to remember also that melatonin is an uncommonly safe molecule when taken as a supplement [72, 79], and it is readily absorbed when it is administered via any route and it crosses easily all morphophysiological barriers, such as the placenta or blood brain barrier [80–82].

Herein, we provide first a brief overview on the growing importance of melatonin in inflammatory-mediated pathological conditions, and thereafter, we identify its protective effects against the activation of inflammasomes, inflammatory intracellular multiprotein complexes. In particular, we focus attention on melatonin interactions with NLRP3 inflammasome, the most widely known and studied inflammasome.

## 2. Melatonin and Inflammation

Inflammation is defined, for many years, as the response to tissue injury and infection. This concept was recently updated due to the avalanche of reports in which molecules and cells associated with inflammation are involved also in the absence of tissue injury or infection. Thus, inflammation may be defined as the innate response to harmful stimuli such pathogens, injury, and metabolic stress, and its ultimate function is to restore the physiological homeostatic state [83]. However, the exact aetiology leading to the development of inflammation is not known, but a combination of genetic, epigenetic, and environmental factors seems to play an important role [84–86]. Initial stages of inflammation are mediated by the activation of the immune system and usually persist for only a short time. However, in this stage, the response may continue and progress to chronic inflammation, which predisposes to various chronic pathologies [45].

Pathological inflammation has been related to several different diseases, and current anti-inflammatory drugs have significant side effects; so, it is important to identify new therapeutic approaches to reduce inflammatory processes without impairing the physiologic inflammatory response. As reported above, melatonin has many actions [14, 37, 43, 87–89] and numerous experimental studies have shown that melatonin is not only an important antioxidant but also a widespread anti-inflammatory molecule [45, 82, 88, 90–92]. Thus, melatonin is a molecule of high clinical interest.

During the inflammatory process, the stimulation of inflammation-related genes may occur due to the activation of the nuclear transcription factor-kappa B (NF-*κ*B) [93]. NF-*κ*B is a ubiquitous oxidative stress-sensitive transcription factor that plays a critical role in the regulation of a variety of genes important in cellular responses including inflammation, innate immunity, cell growth, and death. NF-*κ*B is primarily a cytoplasmic factor that is expressed virtually in all cells and constitutes the major inducible transcription factor whose modulation triggers a cascade of molecular events, some of which are potential key targets for the treatment of inflammation. After its activation, NF-*κ*B translocates to the nucleus and binds to specific elements modulating transcription of proinflammatory genes [45, 94]. Various studies have shown that melatonin modulates the NF-*κ*B signalling pathway during inflammation, and this modulation can occur during early as well as late stages of pathological processes [45, 93, 95–98].

Additionally, cytokines are major mediators of local and intercellular communication required for an integrated response to a variety of stimuli during immune and inflammatory processes. Different cytokines are associated with inflammatory pathological processes; in fact, the clinical outcome is partly determined by the loss of balance between proinflammatory cytokines, like interleukin- (IL-) 1*β*, IL-2, IL-6, interferon-*γ* (IFN-*γ*), and tumor necrosis factor-*α* (TNF-*α*), and anti-inflammatory molecules, such as IL-10 and tumor growth factor-*β* (TGF-*β*) [45, 99–101].

Experimental evidence suggests that melatonin exerts its anti-inflammatory effects by modulating both pro- and anti-inflammatory cytokines in different pathophysiological conditions [45, 102–104]. Furthermore, Carrillo-Vico et al. [105] showed that the presence of melatonin's receptors in a mast cell line modulate an anti-inflammatory pathway via inhibition of TNF-*α* release. Other anti-inflammatory actions of melatonin, and its metabolites AFMK and AMK, are inhibition of prostaglandins synthesis, production of adhesion molecules [106, 107], and downregulation of cyclooxygenase 2 expression in macrophages [37], of leukocyte-endothelial adhesion, and of leukocyte transendothelial cell migration [93, 108], together with the reduction of the polymorphonuclear cell recruitment to the inflammatory site [107, 109]. Furthermore, the overproduction of reactive oxygen species (ROS) contributes significantly to inflammation, a process that leads in turn to the further production of ROS and activation of prooxidant enzymes [45, 88, 93]. Melatonin also counteracts inflammatory processes by scavenging free radicals and activating endogenous antioxidant defense due to its important and well-known antioxidant properties [12, 110–114].

In general, melatonin is highly effective in protecting cells from damage under severe inflammatory conditions [32, 45, 53, 115, 116]. There are several sites where melatonin may interfere during the inflammatory process. As a result, definition of the role of melatonin in the pathophysiological mechanisms of inflammation is a large and growing research field, in which further studies are necessary to elucidate its complex regulatory mechanisms of action in different cells, tissues, and pathological conditions.

## 3. Melatonin and Inflammasomes

As reported above, inflammation is a key feature in the pathogenesis of many clinical conditions [117]. It is also a factor now recognized as a component of aging since this is associated with a low level of inflammatory process, so-called inflammaging [118]; this results from the innate immune system deregulation, chronic proinflammatory cytokine production [119], NF-*κ*B translocation and activation [120], and oxidative stress [121]. Recent studies suggest also a role of the inflammasome in the pathogenesis of different inflammatory diseases [122–125]. Inflammasomes, present in a variety of cells, are intracellular multiprotein complexes that mediate activation of inflammatory caspases [124, 126–128] and in turn induce the secretion of proinflammatory cytokines (IL-1*β* and IL-18) [124, 129, 130]. Inflammasome activation is induced by a variety of endogenous and exogenous signals; in fact, in addition to responding to insults and pathogens, inflammasomes can also be activated by signals stemming from the commensal microbiota [131].

**Figure 1:**
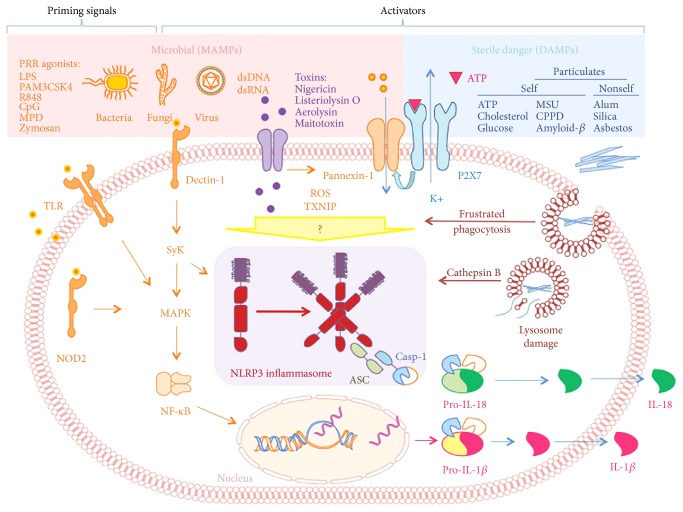
NLRP3 inflammasome activation pathways. ASC: apoptotic-associated speck-like protein containing a caspase recruitment domain; CPPD: calcium pyrophosphate dihydrate; DAMP: danger-associated molecular pattern; LPS: lipopolysaccharide; MDP: muramyl dipeptide; MAMP: microbial-associated molecular pattern; MSU: monosodium urate; ROS: reactive oxygen species; TXNIP: thioredoxin-interacting protein; Casp-1: caspase-1. From Conforti-Andreoni et al. [126] (reprinted by permission—Cellular & Molecular Immunology license number 4125310130103).

To date, five different inflammasomes (nucleotide-binding oligomerization domain-like receptor pyrin domain-containing (NLRP) 1–3) have been clearly identified: NLRP1, NLRP2, NLRP3, AIM2, and IPAF/NLRC4; of these, NLRP3 is the best characterized [132] (Figure 1()).

**Figure 2:**
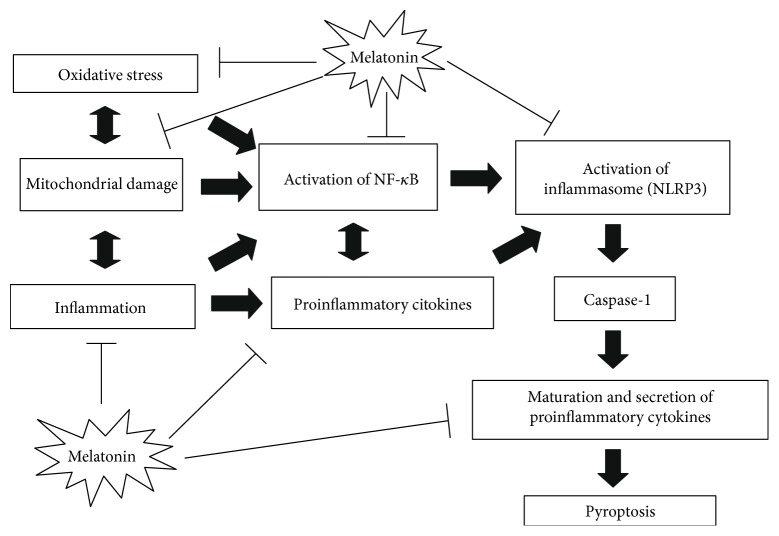
Schematic representation of melatonin's protective effects against the inflammatory process involving NF-*κ*B and NLRP3 inflammasome activation.

The present review focuses on recent insights into the melatonin modulation of the expression of these inflammatory mediators and their effects on cell signalling pathways responsible for melatonin's anti-inflammatory activity. There are relatively few studies about the interaction between melatonin and the inflammasome NLRP3. In detail, NLRP3 inflammasome responds to a variety of signals that are indicative of damage to the host, including environmental irritants, endogenous danger signals, pathogens, mitochondria-derived ROS [133, 134], and DNA released from damaged mitochondria into the cytosol [135]. Activation of the NLRP3 inflammasome requires two signals: microbial molecules or endogenous cytokines, which upregulate the expression of NLRP3 and pro-IL-1*β* through the activation of NF-*κ*B pathway. Thus, the activation of NLRP3 inflammasome-related innate immunity pathways amplifies the inflammatory response NF-*κ*B mediated [120]. The second signal is induced by various damage-associated molecular patterns (DAMPs), leading to the assembly of the inflammasome multiprotein complex [136]. Once the inflammasome is activated, caspase-1 advances pro-IL-1*β* and pro-IL-18 into their mature active process and induces their subsequent secretion [134]. Finally, IL-1*β* and IL-18 initiate an inflammatory process of regulated cell death known as pyroptosis [137]. The activation of the NLRP3 inflammasome can help the host defense against invading bacteria and pathogens; however, excessive activation of the inflammasome can lead to inflammation-associated tissue injury [138] (Figure 2()). The ability of NLRP3 to respond to a variety of signals contributes to its biological importance in several complex diseases, including cancer [139], atherosclerosis [140], type 2 diabetes mellitus [141], gout and metabolic syndrome [142], cardiovascular diseases [143], renal disorders [144], and lung [145] and central nervous [82] diseases.

Infectious aetiologies, such as sepsis and pneumonia, are leading causes of acute lung injury [145–147]. Extracellular histones are produced during acute lung injury and activate the NLRP3 inflammasome promoting in turn the recruitment of neutrophils and suggesting a positive feedback and a potential mechanism of inflammatory propagation [145, 148]. Recently, Zhang et al. [145] demonstrated that intratracheally administration of melatonin reduced pulmonary inflammation in acute lung injury through inhibiting the assembly of the NLRP3 inflammasome. This inhibition is mediated not only through reduction of the release of extracellular histones in the lung but also through direct blockage of extracellular histone-induced activation of the NLRP3 inflammasome. However, the authors reported no alteration in the levels of TNF-*α* expression suggesting that melatonin selectively affects the activation of NLRP3 inflammasome. The inhibition mechanism of the NLRP3 inflammasome activation by melatonin in acute lung injury requires further study.

**Figure 3:**
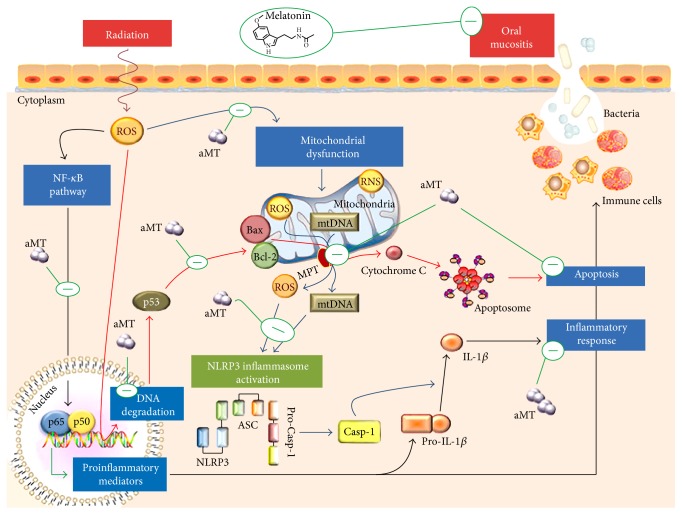
Melatonin protective effects against the development of oral mucositis. In detail, melatonin gel may defend mitochondria and inhibit NF-*κ*B and NLRP3 inflammasome pathways, reducing inflammation and apoptosis. From Ortiz et al. [150] (reprinted by permission—Journal of Pineal Research license number 4123021400967).

Furthermore, other recent publications report on the effect of melatonin treatment against NLRP3 complex assembly in radiation-induced mucositis, sepsis, and aging. Fernández-Gil et al. [149] reported, during mucositis development following tongue irradiation, the involvement of mitochondrial oxidative stress and of bioenergetic impairment in promoting inflammation through the assembly of the NLRP3 inflammasome that leads in turn to caspase-1 activation and cleavage into the mature forms of pro-IL-1*β*, which is produced by NF-*κ*B pathway. Furthermore, its significant increase in the tissue levels of IL-1*β*, TNF-*α*, and cyclooxygenase-2 expression was observed, supporting the hypothesis that NF-*κ*B and NLRP3 work together to activate inflammatory pathways of the innate immune response [149, 150]. Interestingly, the application of melatonin gel prevented mucosal disruption and ulcer formation, protecting the mitochondria from radiation damage and blunting NF-*κ*B/NLRP3 inflammasome signalling activation in the tongue. Ortiz and colleagues [150] obtained interesting results about the melatonin's antagonist effects on both NF-*κ*B and NLRP3 signalling. The therapeutic benefit of melatonin gel against oral mucositis is related to its inhibitory effects on NF-*κ*B/NLRP3 signalling and also on modulation of a correct balance between pro- and anti-inflammatory cytokines; moreover, it may also be attributed to melatonin's capabilities to protect the mitochondria in epithelial cells, as well as stem cells, from radiation. Ortiz and colleagues [150] also showed, for the first time, that melatonin is synthesized in rat tongue, where it acts in autocrine/paracrine signalling by binding to MT1, MT2, and ROR*γ* receptors, suggesting a role of melatonin also in healthy oral mucosal physiology. However, irradiation drastically decreased basal melatonin levels, probably due to its rapid use as a scavenger of free radicals that were induced by radiation. Interestingly, the induction of MT1/MT2 melatonin membrane receptor expression after high-dose melatonin administration indicates that the therapeutic benefits of melatonin are not restricted to low doses. It is important to underline also that systemic administration of melatonin did not achieve effective concentrations in the oral mucosa, so the above-reported observations suggest that the melatonin gel formulation may have important therapeutic potential in patients with oral mucositis (Figure 3). However, it is important to evaluate in depth the mechanisms of action of melatonin together with the dose and modality of administration.

In addition to NLRP3 functions in epithelial cells of the mucosal surface, the inflammasome is also related to the secretion of proinflammatory cytokines that contribute to obesity-associated chronic inflammatory conditions [151–154]. Obesity may induce NLRP3-dependent caspase-1 activation and thus pyroptosis and the proinflammatory response in hypertrophic adipocytes [153, 155]. Recently, Liu et al. [153] demonstrated that exogenous melatonin ameliorates inflammation of adipose tissue by inhibiting the expression of inflammasome genes including NLRP3, apoptotic-associated speck-like protein containing a caspase recruitment domain (ASC), and thereby caspase-1 and IL-1*β*. Furthermore, melatonin in the adipose tissue reduces the phosphorylation of NF-*κ*B and inhibits the NLRP3 pathway in downstream. These findings, with others in which NF-*κ*B signal is central for performing the melatonin's anti-inflammatory functions [153, 156], indicate that melatonin is a potential antiobesity agent that may also reverse obesity-related systemic inflammation.

The anti-inflammatory properties of melatonin have been extensively studied also in models of sepsis in which melatonin inhibits the NF-*κ*B pathway activation [157–159], induces the conservation of the mitochondrial homeostasis, and reduces ROS and proinflammatory cytokine production [125, 159–162]. García et al. [156], Rahim et al. [159], and Volt et al. [120] recently showed that melatonin blunts the NF-*κ*B/NLRP3 connection and activation during cardiac sepsis and aging. These studies provide the basis for further investigations on the efficacy of melatonin in protecting against myocardial damage and in enhancing the survival of septic mice [159, 163, 164]. Thus, the NLRP3 inflammasome could be considered as a target of melatonin, which transforms a severe myocardial septic condition into a moderate inflammatory state, thus preventing cardiac failure [159, 163]. However, the specific interaction of melatonin with the NLRP3 inflammasome during sepsis in cardiac tissue remains to be clearly identified. Furthermore, to test the anti-inflammatory beneficial effects of melatonin, Rahim and colleagues carried out a phase II clinical trial in septic patients (Eudract number 2008-006782-83) who were treated with a new-patented injectable formulation of melatonin (PCT/ES2015070236) and the preliminary data related to this clinical trial suggested the same beneficial effects of melatonin in humans as observed in experimental animal models.

Chronic melatonin treatment absolutely prevented age-dependent oxidative stress and mitochondrial impairment in multiple organs including the heart [120, 165, 166]. Moreover, Volt et al. [120] showed that melatonin administration in aged mice counteracted the septic response, reducing inflammation, oxidative stress, and mitochondrial alterations and inhibiting the intrinsic apoptotic pathway; however, melatonin did not counteract the inflammaging response. These results account for an important duality of melatonin effects: chronic, low doses of melatonin prevent the age-dependent increase in inflammation, ROS, and mitochondrial impairment characteristic of inflammaging [165–169]; by comparison, acute, high doses of melatonin counteract septic inflammation without influencing the basal inflammatory status of aged mice. According to Volt et al. [120], melatonin should be administered in a chronic schedule in order to prevent inflammaging and in acute schedule to counteract severe inflammatory responses. Eventually, further investigations are necessary to clarify whether the decreased melatonin production [56, 66, 170] promotes the chronoinflammaging process. Also, Garcia et al. [156] evaluated the melatonin effect against NF-*κ*B/NLRP3 inflammatory pathway in mouse heart during sepsis, demonstrating for the first time that melatonin requires the ROR*α* receptors to blunt the NF-*κ*B/NLRP3 connection during sepsis. The multiple molecular targets of melatonin observed in the reported studies explain and justify its potent anti-inflammatory efficacy against systemic innate immune activation and underline a promising melatonin therapeutic application in the treatment of sepsis.

The beneficial effects of melatonin due to the inhibition of the NLRP3 inflammasome have received increased attention since it is related to the improvement of many inflammatory diseases [82, 145, 159, 171]. In this regard, Cao et al. [171] showed that cadmium exposure significantly increased the expression of NLRP3 and active caspase-1 (p20) in the liver, suggesting that NLRP3 is involved in cadmium-induced hepatocyte death and that this type of hepatocyte death is caspase-1 dependent. Interestingly, the authors found that melatonin treatment significantly alleviated cadmium-induced liver injury by decreasing serum alanine aminotransferase/aspartate aminotransferase levels, suppressing the production of proinflammatory cytokines (IL-1*β*, TNF-*α*, and IL-6) and the induction of NLRP3 mRNA and protein expression. Cao et al. [171] documented that the protective effect of melatonin is mediated by the thioredoxin-interacting protein- (TXNIP-) NLRP3 inflammasome pathway. Thus, TXNIP is a key endogenous regulator of the cellular redox balance that plays an important role in the pathogenesis of acute liver failure and directly activates the NLRP3 inflammasome under oxidative stress conditions [171, 172]. Targeting TXNIP-NLRP3 pathway may represent a therapeutic approach for treating liver disease [171, 173].

NLRP3 inflammasome is associated also with the incidence and progression of central nervous system diseases including cerebral hemorrhage [174], ischemic stroke [175], meningitis [176], Parkinson disease [177], glutamate-associated brain damage [178], and subarachnoid hemorrhage [82, 138]. Recently Dong and collaborators [82] demonstrated that melatonin exerts neuroprotective actions via its antiapoptotic effects [179, 180], which are NLRP3 inflammasome-associated. To summarize, Dong et al. [82] observed that melatonin has neuroprotective properties against subarachnoid haemorrhage, inducing increased survival rate, as well as elevated neurological scores and brain antioxidant content and reduced brain oedema, apoptotic ratio, and blood brain barrier disruption. These actions involve inhibition of NLRP3 inflammasome activation and the subsequent production of IL-1*β* and IL-18.

A novel melatonin derivative, 5-hydroxy-2′-isobutyl-strepchlorin (HIS) was recently synthesized from deacetylmelatonin. In their observations, Shim and collaborators [181] stated that low concentrations of HIS significantly inhibited the NLRP3 inflammasome activation by interference with formation of NLRP3 inflammasome complex without mitochondrial ROS inhibition.

## 4. Conclusion

Actually, NLRP3 inflammasome and/or mitochondria are known to be major targets of interest for the pharmacological management of inflammatory diseases. Recent years have seen significant progress in understanding how inflammasomes contribute to the pathology of multiple inflammatory and immune diseases [123, 124, 171]. However, many important questions remain to be answered, including how host cells determine which inflammasome activates under specific conditions and how inflammasome signalling is interwoven with other innate and adaptive immune pathways. In summary, the multiple molecular targets of melatonin observed in the reported studies explain its potent anti-inflammatory efficacy against systemic innate immune activation and underline a promising therapeutic application for melatonin in the treatment of different inflammatory-mediated pathological conditions.
